# Systems Pharmacology-Based Strategy to Investigate Pharmacological Mechanisms of Total Flavonoids in *Dracocephalum moldavica* on Chronic Heart Failure

**DOI:** 10.3390/ijms23158409

**Published:** 2022-07-29

**Authors:** Awaguli Dawuti, Shuchan Sun, Ranran Wang, Difei Gong, Tianyi Yuan, Li Zhang, Shiying Yang, Jianguo Xing, Ruifang Zheng, Yang Lu, Shoubao Wang, Lianhua Fang, Guanhua Du

**Affiliations:** 1 State Key Laboratory of Bioactive Substances and Functions of Natural Medicines, Institute of Materia Medica, Chinese Academy of Medical Sciences and Peking Union Medical College, Beijing 100050, China; awaguli@imm.ac.cn (A.D.); sunsc@imm.ac.cn (S.S.); wangranran@imm.ac.cn (R.W.); gongdf@imm.ac.cn (D.G.); yuantianyi@imm.ac.cn (T.Y.); luy@imm.ac.cn (Y.L.); 2 Beijing Key Laboratory of Drug Targets Identification and Drug Screening, Institute of Materia Medica, Chinese Academy of Medical Sciences and Peking Union Medical College, Beijing 100050, China; shoubaowang@imm.ac.cn; 3 Beijing Key Laboratory of Polymorphic Drugs, Institute of Materia Medica, Chinese Academy of Medical Sciences and Peking Union Medical College, Beijing 100050, China; zhangl@imm.ac.cn (L.Z.); ysy@imm.ac.cn (S.Y.); 4 Xinjiang Key Laboratory of Uygur Medical Research, Xinjiang Institute of Materia Medica, Urumqi 830004, China; xjguodd@163.com (J.X.); zrfangdd@163.com (R.Z.)

**Keywords:** heart failure, flavonoids extracted from *Dracocephalum moldavica* L., systems pharmacology, molecular docking, HFpEF, inflammation

## Abstract

Heart failure (HF) is a clinical syndrome of cardiac insufficiency caused by abnormalities in cardiac structure and function that arise for various reasons, and it is the final stage of most cardiovascular diseases’ progression. Total flavonoid extract from *Dracocephalum moldavica* L. (TFDM) has many pharmacological and biological roles, such as cardioprotective, neuroprotective, anti-atherogenic, antihypertensive, anti-diabetic, anti-inflammatory, antioxidant, etc. However, its effect on HF and its molecular mechanism are still unclear. In this study, we used systems pharmacology and an animal model of HF to investigate the cardioprotective effect of TFDM and its molecular mechanism. Eleven compounds in TFDM were obtained from the literature, and 114 overlapping genes related to TFDM and HF were collected from several databases. A PPI network and C-T network were established, and GO enrichment analysis and KEGG pathway analysis were performed. The top targets from the PPI network and C-T network were validated using molecular docking. The pharmacological activity was investigated in an HFpEF (heart failure with preserved ejection fraction) mouse model. This study shows that TFDM has a protective effect on HFpEF, and its protective mechanism may be related to the regulation of proinflammatory cytokines, apoptosis-related genes, fibrosis-related genes, etc. Collectively, this study offers new insights for researchers to understand the protective effect and mechanism of TFDM against HFpEF using a network pharmacology method and a murine model of HFpEF, which suggest that TFDM is a promising therapy for HFpEF in the clinic.

## 1. Introduction

Heart failure (HF) is a complex clinical syndrome caused by structural and/or functional cardiac abnormalities that result in the impairment of ventricular filling and/or ejection, ultimately causing the inability of the heart to supply the peripheral tissues with the required amount of blood and oxygen to satisfy their metabolic demands. Across 60 included studies, 1.5 million people suffered from heart failure [1]. According to the left ventricle ejection fraction (LVEF), patients with heart failure are commonly classified into three groups: heart failure with reduced ejection fraction (HFrEF), heart failure with mildly reduced ejection fraction (HFmrEF), and heart failure with preserved ejection fraction (HFpEF) [2]. Approximately half of the patients with signs and symptoms of heart failure, such as shortness of breath, limited exercise capacity, or cough, have a left ventricular ejection fraction that is not markedly abnormal on echocardiography, indicating that systolic function is normal, which is diagnosed as HFpEF [3]. Long-term mortality and re-hospitalization rates for HFpEF are similar to those for HFrEF [4,5]. Hypertension, hyperlipidemia, type 2 diabetes mellitus (T2DM), and obesity are clinical risk factors for HFpEF [6,7]. The pathophysiological mechanisms responsible for the development of HFpEF have not been fully elucidated. The clinical syndrome of HFpEF can be triggered by a wide range of pathophysiologic changes, including myocardial ischemia, endothelial dysfunction, RAAS (renin–angiotensin aldosterone system) activation, fibrosis, inflammation, reduced cardiomyocyte compliance, cardiomyocyte hypertrophy, and oxidative stress [8]. Currently, there are no FDA-approved medications available for HFpEF [9]. Therefore, it is necessary to find active ingredients that are effective against HFpEF.

Traditional Chinese medicine (TCM) has attracted worldwide attention in recent decades for its precise efficacy, relatively low toxicity, and low cost [10]. TCM has a long history of use in clinical treatment and is characterized by multiple ingredients, multiple targets, and multiple pathways, which may be an effective source for new drug development. *Dracocephalum moldavica* L. (*D. moldavica* L.) is an annual plant belonging to the family Labiatae [11]. It is widely found in different regions, including China, Siberia, Russia, Egypt, and Europe [12]. Total flavonoid extract from *D. moldavica* L. (TFDM) has many pharmacological and biological roles, such as cardioprotective, neuroprotective, anti-atherogenic, antihypertensive, anti-diabetic, anti-inflammatory, antioxidant, etc. [13,14]. The cardioprotective effects of TFDM are mainly expressed as anti-myocardial ischemia–reperfusion injury activity [14,15]. Currently, there are no clinical or experimental reports on whether it has a protective effect on HFpEF.

**Figure 1:**
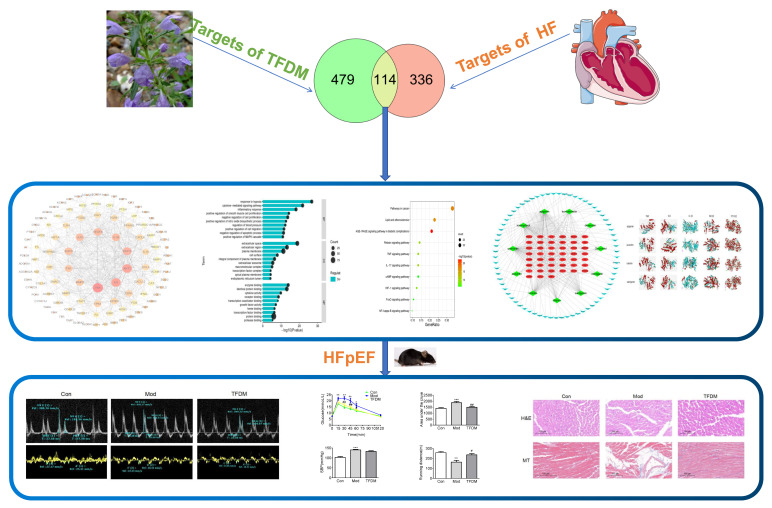
Workflow of the present study.

In this study, we used systems pharmacology to estimate the target compounds of TFDM and targets for heart failure by using several databases. We then constructed PPI and C-T networks and conducted Gene Ontology, KEGG pathway enrichment, and molecular docking analyses for the validation of the top targets. Finally, we used a novel two-hit murine model [16] of HFpEF in which concomitant metabolic and hypertensive stress was elicited by a high-fat diet (HFD) coupled with N[w]-nitro-l-arginine methyl ester (L-NAME) to study the protective effect of TFDM on HFpEF. The whole workflow is illustrated in Figure 1.

## 2. Results

### 2.1. Network Pharmacology-Based Analysis

#### 2.1.1. Component Analysis of TFDM

Through the PubMed, Wanfang, and CNKI databases, 34 kinds of flavonoids were collected from the literature, among which 11 kinds of flavonoids had related information in PubChem, TCMSP, CTD, and SwissTarget Prediction databases: apigenin, acacetin, luteolin, salvigenin, chryseriol, diosmetin, isorhamnetin, kaempferol, quercetin, tilianin, and astragalin. To identify the pharmacokinetic properties of each compound, two classical ADME parameters, OB and DL, were obtained from the TCMSP database. Among them, eight compounds had an OB greater than 30.0%; only apigenin (23.06%), tilianin (19.66%), and astragalin (14.03%) had an OB less than 30.0%. The DL of all flavonoids in TFDM was greater than 0.18 (Table 1). The chemical structures and PubChem CIDs of constituents are shown in Table 2.

#### 2.1.2. Compounds, Disease Target Prediction, and Construction of PPI Network

**Figure 2:**
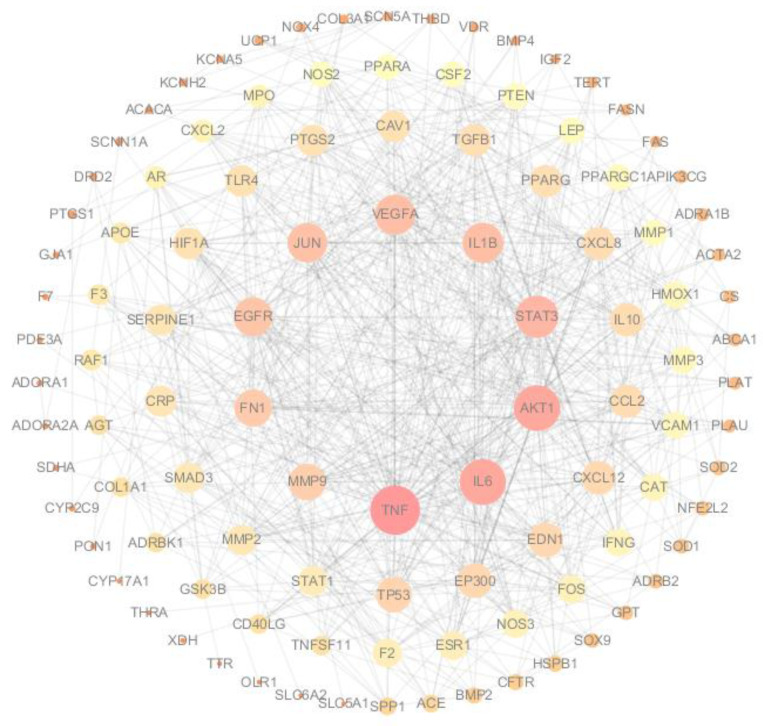
PPI network of common compound–disease targets. The size and color of nodes indicate the magnitude of the degree. A larger size of a node means a larger degree.

To search for the targets of the 11 flavonoids, TCMSP, CTD, and SwissTargetPrediction databases were used, and 593 targets were identified. A total of 450 known therapeutic targets for heart failure were collected from the TTD, Gene Cards, and DisGeNET databases. The 593 compound targets and 450 disease targets have 114 overlaps. This means that these 114 genes may be key targets for the treatment of heart failure with TFDM. To further clarify the correlations among TFDM targets associated with HF targets, a “target-target network” was built based on the STRING results of the 114 overlapping genes. Nodes and edges represent targets and associations between targets, respectively, in the PPI network. The size and color of nodes indicate the magnitude of the degree. A larger-sized node means a larger degree. A total of 106 nodes and 700 edges were involved in the PPI network, as shown in Figure 2. The top 10 targets included TNF, IL6, AKT1, STAT3, IL-1β, and VEGFA, among others.

#### 2.1.3. Construction of C-T Network

**Figure 3:**
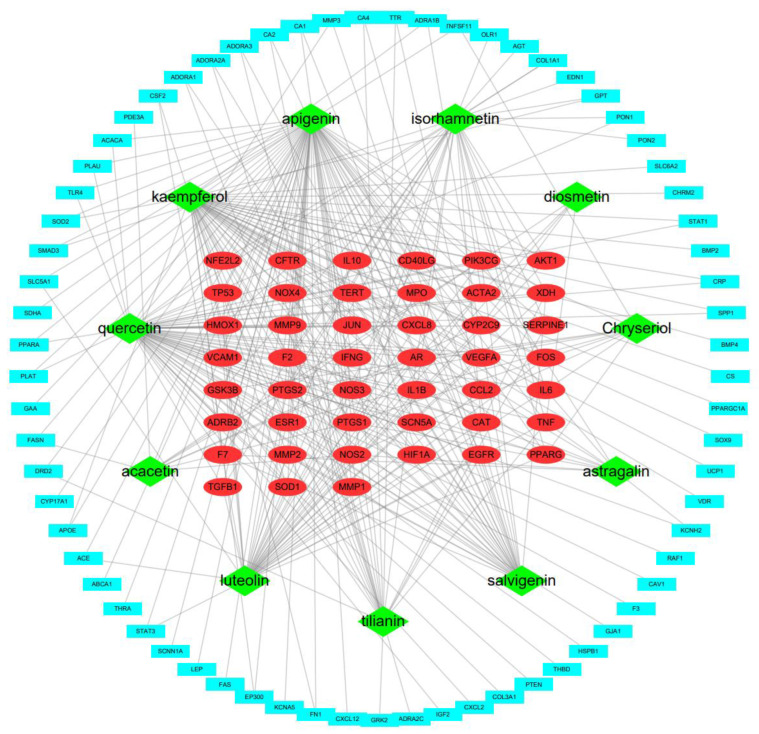
The compound–target network. Green diamonds represent compounds, red ellipses represent targets whose degree is greater than or equal to three, and blue rectangles represent targets whose degree is less than or equal to two.

To uncover the interactions between constituents and potential targets for treating HF, a C-T network based on 11 compounds and 114 potential targets was constructed. The network is composed of 125 nodes and 339 edges, in which green diamonds represent compounds, red ellipses represent targets whose degree is greater than or equal to 3, and blue rectangles represent targets whose degree is less than or equal to 2. An edge represents the interaction between the compound and the target (Figure 3). The higher the node degree, the more important it is in the network. The top 10 targets in node degrees are shown in Table 3.

#### 2.1.4. Gene Ontology Enrichment Analysis

**Figure 4:**
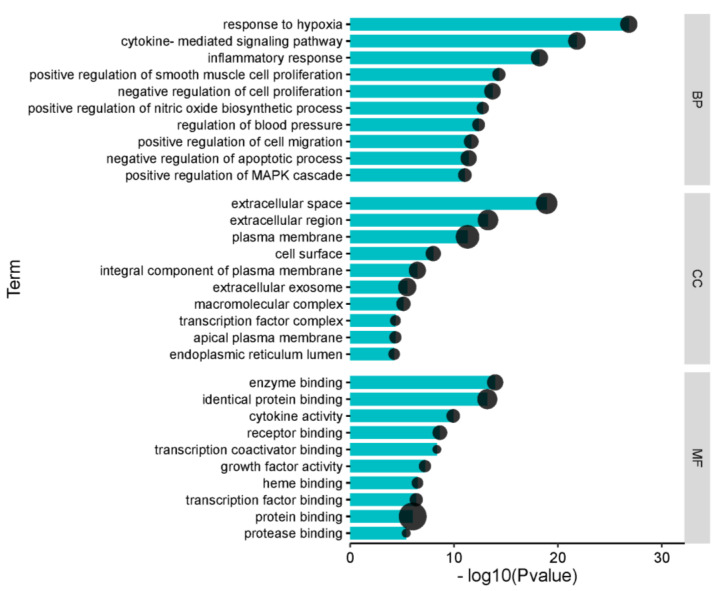
Gene Ontology (GO) analysis of the 114 overlapping gene symbols associated with heart failure. BP represents the categories of “biological process”, CC represents the categories of “cellular component”, and MF represents the categories of “molecular function”. Black nodes represent gene numbers.

To verify whether the 114 genes are related to heart failure, we conducted GO enrichment analysis to clarify the relevant biological processes, cellular components, and molecular functions, as shown in Figure 4. The *Y*-axis represents the GO term. The *X*-axis indicates -Log10 (*p* value). Black nodes represent the number of genes enriched for the term. We used the first 10 terms from small to large according to the *p*-value for a brief demonstration. The results indicate that numerous biological processes are involved in the treatment of heart failure, including response to hypoxia, cytokine-mediated signaling pathway, inflammatory response, negative regulation of cell proliferation, positive regulation of nitric oxide biosynthetic process, regulation of blood pressure, positive regulation of MAPK cascade, and others.

#### 2.1.5. KEGG Pathway Enrichment Analysis

**Figure 5:**
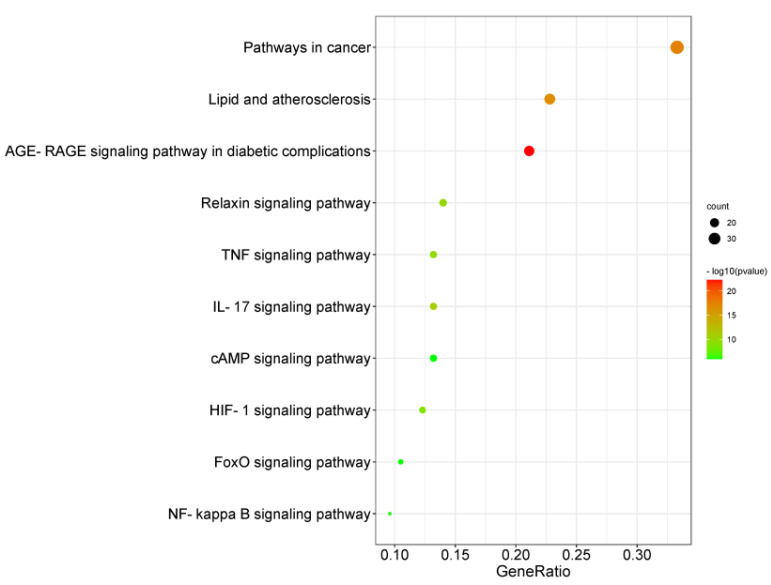
KEGG pathway enrichment of common component–disease targets. The *Y*-axis represents KEGG pathways. The *X*-axis indicates the gene ratio enriched in the pathway.

KEGG pathway enrichment analysis was performed by using the DAVID database, as shown in Figure 5. The *Y*-axis represents KEGG pathways. The *X*-axis indicates the gene ratio enriched in the pathway. The redder the color, the smaller the value of *p*, which also means more credibility and more importance. In this study, we sorted the first 10 pathways from small to large according to the *p*-value for a brief demonstration. Among these pathways, the AGE-RAGE signaling pathway in diabetic complications, lipid and atherosclerosis, IL-17 signaling pathway, relaxin signaling pathway, TNF signaling pathway, HIF-1 signaling pathway, NF-kappa B signaling pathway, FoxO signaling pathway, cAMP signaling pathway are considered the top priority.

#### 2.1.6. Computational Validation of Selected Compound–Target Interactions

**Figure 6:**
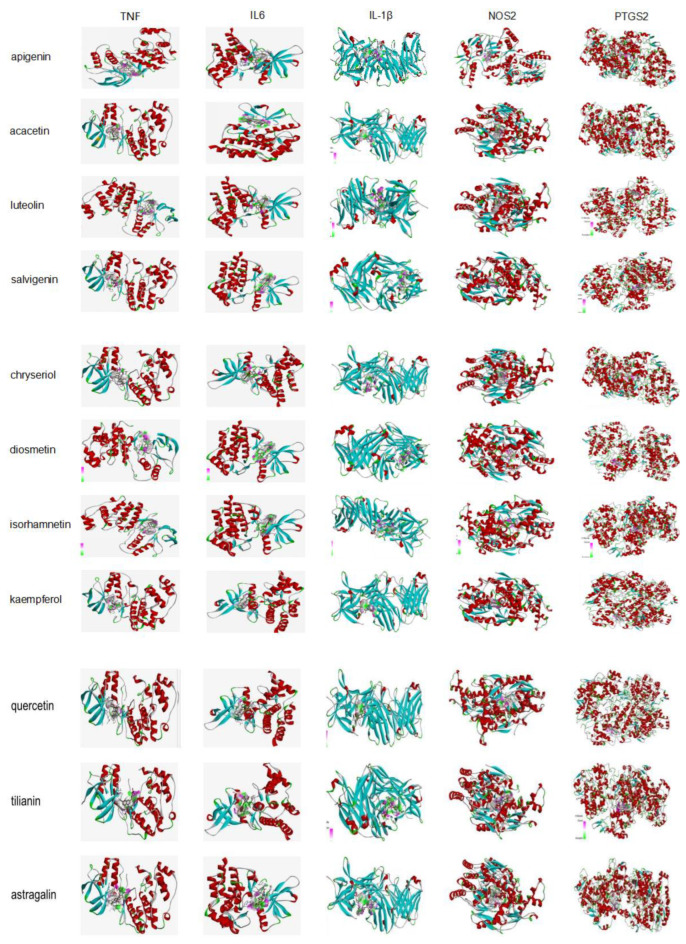
Molecular docking of compounds with top targets.

Eleven compounds from TFDM were analyzed by molecular docking with the top targets: TNF, IL6, IL-1β, NOS2, and PTGS2 in the PPI network and C-T network, respectively. The molecular docking results showed that the binding energies (Vina scores) of all of the compounds with the top five targets were lower than −7.1 (Table 4, Figure 6). This means that these 11 compounds stably bind to these targets and may be key targets for the treatment of heart failure with TFDM.

### 2.2. Effects of TFDM

#### 2.2.1. Effects of TFDM on Blood Glucose, Blood pressure, and Running Distance

**Figure 7:**
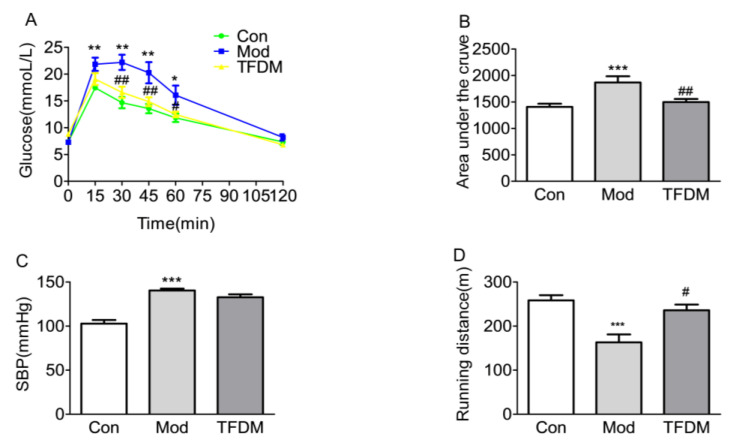
Effects of TFDM on blood glucose, blood pressure, and running distance. (**A**) Intraperitoneal glucose tolerance test (ipGTT) after treatment. (**B**) Bar graphs depicting the area under the curve of 10-week time points in the ipGTT experiment. (**C**) Systolic blood pressure (SBP) of different experimental groups. (**D**) Running distance of different experimental groups (*n* = 6). Compared with Con group: * p < 0.05, ** p < 0.01, *** p < 0.001; compared with Mod group: ^#^ *p* < 0.05, ^##^ *p* < 0.01.

Compared with the normal control group, the model group showed significantly impaired glucose handling. After intraperitoneal injection of 2 g/kg glucose after 6 h fasting, the blood glucose of model group mice increased significantly after 15, 30, 45, and 60 min, while treatment with TFDM significantly reduced impaired glucose handling (Figure 7A,B). Long-term drinking of water with the addition of L-NAME triggered hypertension, but there was no significant difference between the model group and TFDM treatment group, indicating that TFDM has no antihypertensive effect on L-NAME-induced hypertension (Figure 7C). Reduced exercise endurance is one of the main symptoms of heart failure. The results of an exercise exhaustion test showed that the running distance of the model group was significantly reduced, and TFDM treatment significantly improved the fatigue of mice (Figure 7D).

#### 2.2.2. Effects of TFDM on Cardiac Function

**Figure 8:**
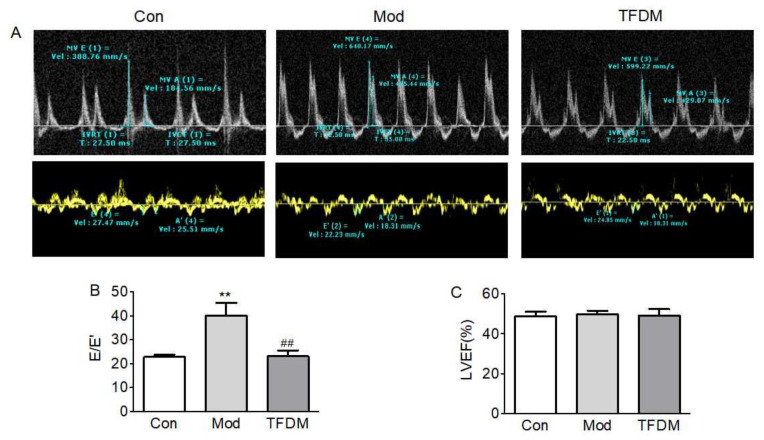
Effects of TFDM on cardiac function. (**A**) Representative images of pulsed-wave and tissue Doppler recording through the left ventricle. (**B**) Ratio between mitral E wave and E’ wave (E/E’). (**C**) Percentage left ventricular ejection fraction (LVEF%) (*n* = 6); compared with Con group: ** *p* < 0.01; compared with Mod group ^##^ *p* < 0.01.

Ten weeks of exposure to HFD plus L-NAME triggered left ventricle diastolic dysfunction but did not affect systolic function (Figure 8C). The E/E’ ratio is a key index to evaluate the left ventricular diastolic function. Compared with the normal control group, the E/E’ ratio of the model group was significantly increased, while that of the TFDM treatment group was significantly decreased, indicating that TFDM treatment has a significant improvement effect on diastolic dysfunction (Figure 8A,B).

#### 2.2.3. Effects of TFDM on Cardiac Histology

**Figure 9:**
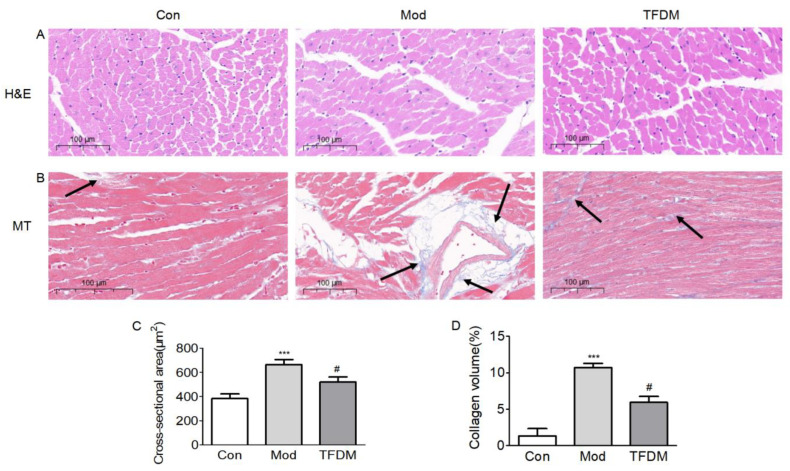
Effects of TFDM on cardiac histology. (**A**) Representative images of hematoxylin and eosin (H&E). (**B**) Representative images of Masson’s Trichrome (MT) staining of transversal sections of the left ventricle of mice. Scale bars: 100 μm. (**C**) The quantification of cardiomyocyte cross-sectional area. (**D**) The quantification of collagen volume. Localisation of collagen fibres demonstrated by black arrow. Three samples in each experimental group and three sections from each individual sample were analyzed by Image J software. Compared with Con group: *** *p* < 0.001; compared with Mod group ^#^ *p* < 0.05.

H&E and Masson staining results revealed that, compared with the normal control group, the myocardium of the model group showed obvious hypertrophy and fibrosis (Figure 9A,B). The model group showed a significant increase in the cross-sectional area (CSA) of cardiomyocytes, while TFDM treatment significantly decreased the CSA (Figure 9C). Masson’s trichome staining showed increased cardiac collagen volume in model group mice, and TFDM treatment significantly decreased the cardiac collagen volume (Figure 9D).

## 3. Discussion

HF is the most rapidly growing cardiovascular health burden worldwide, and approximately half of newly diagnosed HF patients are HFpEF. Furthermore, mortality and morbidity in HFpEF have continuously increased [21]. As the prevalence increases, the social cost of HFpEF greatly increases, but no verified treatment regimen is available [22]. Many treatments that are effective for HFrEF failed to improve the symptoms and prognosis in HFpEF patients [23]. For this reason, there is a great need to find effective drugs for HFpEF. HF is related to multiple risk factors, and its pathogenesis has not been elucidated yet. Therefore, multitarget treatments may be more effective in treating HF. TCM is characterized by multiple compounds, multiple pathways, and multiple targets. *D. moldavica* L. is one kind of traditional medicine, and TFDM is the total flavonoids extracted from *D. moldavica* L. In this study, we used several databases to collect 11 known monomeric compounds identified in TFDM, namely, apigenin, acacetin, luteolin, salvigenin, chryseriol, diosmetin, isorhamnetin, kaempferol, quercetin, tilianin, and astragalin. Our study aimed to evaluate the effects and mechanisms of the constituents of TFDM for the treatment of HFpEF. According to the target distribution of constituents in TFDM related to HF, the inflammatory reaction is the main target that TFDM affects, and TNF, IL6, and IL-1β are the major targets of the constituents of TFDM linked to the potential treatment of HF. The results of KEGG pathway enrichment analysis also demonstrate that the therapeutic effect of TFDM on HF may be achieved by inhibiting the IL-17 signaling pathway, TNF signaling pathway, NF-kappa B signaling pathway, etc. Inflammation is accepted as an important pathophysiological factor in HF. Specifically, inflammation has been linked to disease development, progression, and complications [24]. Although inflammation contributes to the pathogenesis and progression of HF across the spectrum of both HFrEF and HFpEF, a stronger association with inflammatory markers may exist in the context of HFpEF [25]. TNF-α, IL-1β, IL-6, IL-17, IFN-γ, and IL-18 signaling molecules can induce both hypertrophy and apoptosis and induce further inflammation in the setting of cardiac injury [26]. TFDM shows protective effects against atherosclerosis by resisting inflammatory reactions [27]. Tilianin derived from TFDM ameliorates atherosclerosis by inhibiting the production of the NF-κB-dependent proinflammatory cytokines TNF-α and IL-1β via the inhibition of IκB kinase activity [28]. Apigenin improves hypertension and cardiac hypertrophy in spontaneously hypertensive rats, which are associated with the downregulation of inflammatory factors, such as IL-1β, IL-6, and iNOS in the hypothalamic paraventricular nucleus [29]. Kaempferol remarkably decreased inflammation and oxidative stress in Ang II-stimulated cardiac fibroblasts by modulating AMPK/Nrf2 and NF-κB pathways [30]. Luteolin protects heart tissues from STZ-induced [31], cobalt-induced [32], and LPS-induced [33] inflammation by suppressing the NF-кB signaling pathway. Quercetin treatment can inhibit the inflammatory response, oxidative stress, and cell apoptosis in an oxygen-glucose deprivation model of H9C2 cells [34]. These studies suggest that the cardioprotective effects of TFDM are mainly related to its anti-inflammatory effects.

PTGS2, namely, COX-2, and NOS2, namely, iNOS, were also among the main predicted targets in our study, and they play an important role in HF. Clinical data suggest that a systemic imbalance in NO levels and inflammation are crucial in the development of HFpEF [35]. In our experimental model, L-NAME, which we used as a driver of endothelial dysfunction-based hypertension, is a more potent inhibitor of eNOS and nNOS as compared to iNOS [36]. In the cardiovascular system, L-NAME-induced events are associated with the upregulation of iNOS [37]. Similarly, in rodents exposed to HFD, iNOS is upregulated [38]. Other findings also demonstrate that meta-inflammation and its master mediator, iNOS, are critical elements in the pathophysiology of HFpEF [16]. In our present study, the C-T network analysis showed that iNOS can be linked to 10 compounds in TFDM, which reflects that the effect of TFDM on HFpEF may be realized by inhibiting iNOS, and TCM works through multiple active compounds acting on a single target.

Cardiomyocyte death is a common characteristic of heart injury. Apoptosis is the principal cellular pathway that results in cardiomyocyte death. In our study, the PPI and C-T network analysis showed that PIK3CG, namely, PI3K, and AKT are also among the main predicted targets through which TFDM exerts its protective effects on HF. Consistent with our research, other studies also showed that TFDM [14] and tilianin [39] pretreatment attenuates ischemia–reperfusion-induced apoptosis via regulating the PI3K/Akt signaling pathways.

Network pharmacology is a useful tool and strategy to predict potential therapeutic mechanisms and can effectively provide research directions [40]. With the help of network pharmacology, it is possible to elucidate the complex molecular mechanisms underlying TCM and further build novel therapeutic applications beyond the traditional TCM application [41]. Taking into account the possible spurious associations between one database and another, it is necessary to verify the results. Therefore, our investigation applied in vivo experiments to build an HFpEF mouse model, verified the protective effect of TFDM on HFpEF, and confirmed the predicted targets by using molecular docking.

In our experimental study, the echocardiogram results showed that TFDM significantly reduced the E/E’ ratio. In diastolic dysfunction, the E/E’ ratio is markedly increased, and it is one of the criteria used in the differential diagnosis [22]. The severity of glucose intolerance in patients with HF correlates with the functional and clinical severity of HF [42]. Our in vivo experiments revealed that treatment with TFDM significantly reduced HFD-induced glucose handling impairment. KEGG pathway enrichment analysis also showed that the effect of TFDM on HF may be related to the AGE-RAGE signaling pathway in diabetic complications. Cardiac fibrosis provokes pathological changes that culminate in chamber dilatation, cardiomyocyte hypertrophy, and apoptosis and ultimately lead to the development of HF [43,44]. The H&E and Masson staining results reveal that TFDM treatment can improve HFD- and L-NAME-induced cardiomyocyte hypertrophy and cardiac fibrosis. TFDM not only improved the pathological structure of the heart but also increased the limited exercise capacity of HFpEF mice.

Molecular docking is an established in silico structure-based method widely used in drug discovery. Docking enables the identification of novel compounds of therapeutic interest by predicting ligand–target interactions at a molecular level [45]. AutoDock Vina is one of the fastest and most widely used open-source programs for molecular docking [46]. In this study, eleven compounds from TFDM were analyzed by molecular docking with the top targets selected from the PPI network and C-T network. The results demonstrated that all 11 compounds from TFDM had good binding activities to important targets. It is generally considered that the value of the Vina score indicates a certain binding activity between a protein and a compound, and the more negative the binding energy, the more stable the binding of the compound to the target [47].

In summary, our research systematically investigates TFDM from a whole action mechanism perspective in the treatment of HF, specifically in the treatment of HFpEF, and provides a basis for exploring multi-compound synergies in subsequent research. TFDM can realize the protective effect of HF through multiple targets, multiple biological processes, and multiple signaling pathways. A total of 11 compounds in TFDM were obtained from the literature, and 114 overlapping genes between TFDM and HF were screened using systems pharmacology combined with target predictions. The PPI network, C-T network, GO enrichment analysis, and KEGG pathway analysis suggested that the pharmacological mechanism of TFDM for the treatment of HF may be related to its involvement in the regulation of proinflammatory cytokines, apoptosis-related genes, fibrosis-related genes, etc. At the end of our research, the protective effect of TFDM was confirmed in an HFpEF mouse model. In general, this study provides a comprehensive understanding of the mechanism of TFDM for the treatment of HFpEF.

## 4. Materials and Methods

### 4.1. Network Pharmacology-Based Approach for the Potential Actions of TFDM on HF

#### 4.1.1. Screening of Active Compounds

All compounds contained in TFDM were identified from the literature by using the PubMed database, Wanfang database, and China National Knowledge Infrastructure (CNKI) database, and then the pharmacokinetic properties of each compound, such as oral bioavailability (OB) and drug-likeness (DL), were obtained from the Traditional Chinese Medicine Systems Pharmacology database (TCMSP, https://old.tcmsp-e.com/tcmsp.php (accessed on 7 November 2021)). OB represents the percentage of an orally administered dose of unchanged drug that reaches the systemic circulation. High oral bioavailability is often a key indicator to determine the drug-like properties of bioactive molecules as therapeutic agents [48]. DL is a qualitative parameter used in drug design to estimate how “drug-like” a prospective compound is, which helps to optimize pharmacokinetic and pharmaceutical properties. The “drug-like” level of the compounds is 0.18, which is used as a selection criterion for “drug-like” compounds in traditional Chinese herbs [49].

#### 4.1.2. Target Prediction

Targets of the compounds in TFDM were retrieved from TCMSP, CTD (http://ctdbase.org/ (accessed on 8 November 2021)), and SwissTargetPrediction (http://swisstargetprediction.ch (accessed on 8 November 2021)) databases, and the target name was then transformed to the gene symbol using the UniProt knowledge database. To collect the disease targets for heart failure, we searched with the keywords “heart failure” or “chronic heart failure” in TTD (http://db.idrblab.net/ttd/ (accessed on 8 November 2021)), Gene Cards (https://www.genecards.org/ (accessed on 8 November 2021)), and DisGeNET (https://www.disgenet.org/home/ (accessed on 8 November 2021)) databases, and duplicated disease targets were removed. Finally, Draw Venn Diagram was used to analyze the target intersection between heart failure and TFDM.

#### 4.1.3. Protein–Protein Interaction (PPI) Network and Compound–Target (C-T) Network Construction

The target intersection between HF and TFDM was imported into the STRING database (https://string-db.org/ (accessed on 9 November 2021)) to perform PPI analysis. The organism was specified as Homo sapiens, and proteins corresponding to genes with high confidence (minimum required interaction score ≥ 0.7) were selected. The results were saved in TSV file format and imported into Cytoscape software (v.3.7.1, National Institute of General Medical Sciences, San Diego, CA, USA) to visualize the PPI network. To evaluate the therapeutic mechanism of TFDM in treating HF, the compound–target (C-T) network was constructed by using Cytoscape 3.7.1 software.

#### 4.1.4. Gene Ontology, KEGG Pathway Enrichment, and C-T Network Construction

To explore the possible biological processes (BPs), cellular components (CCs), molecular functions (MFs), and biological pathways of common compound–disease targets, Gene Ontology and KEGG pathway enrichment analyses were performed on the DAVID (https://david.ncifcrf.gov/ (accessed on 10 November 2021)) database.

#### 4.1.5. Target Identification Based on Molecular Docking

The three-dimensional (3D) structures of the five targets were downloaded from the RCSB PDB database (https://www.rcsb.org/ (accessed on 13 November 2021)). The PubChem database (https://pubchem.ncbi.nlm.nih.gov/ (accessed on 13 November 2021)) was used to download the two-dimensional (2D) structures of the eleven compounds. AutoDock Tools software (v.1.5.6, Center for Computational Structural Biology, La Jolla, CA, USA) was used to remove water molecules, isolate proteins, add nonpolar hydrogen molecules, and calculate Gasteiger charges for the structures, which were saved as a PDBQT file. The active site of molecular docking was determined by the ligand coordinates in the target protein complex.

### 4.2. In Vivo Experiments to Validate the Effect of TFDM on HF

#### 4.2.1. Reagents and Animals

TFDM was provided by Xinjiang Institute of Materia Medica (purity 57%, batch no. 20100626). HFD (60% calories from lard, D12492, Research Diet) and L-NAME (Sigma Aldrich) were used for the model. Eight–ten-week-old male mice (*n* = 10/group), were purchased from Vital River Laboratories, Beijing, China. Animal care and handling protocols were approved by the Animal Ethics Committee of the Chinese Academy of Medical Science and Peking Union Medical College.

#### 4.2.2. Establishment of HFpEF Experimental Model

Mice with similar baseline characteristics were randomly divided in to 3 groups (*n* = 10 per group): the control group, model group, and TFDM group. Mice in the model group and TFDM group were fed a high-fat diet and water, and L-NAME (0.5 g/L) was supplied in the drinking water for ten weeks after adjusting the pH to 7.4. Mice in the control group were fed a CHOW diet and drinking water without L-NAME. At Week 6, TFDM (90 mg/kg, suspended in 0.5% CMC-Na) was orally administered every day for 28 days. The control and model groups received the same volume of saline.

#### 4.2.3. Conventional Echocardiography and Doppler Imaging

Three days prior to sacrifice, echocardiography was performed with the Visual Sonics Vevo 770 system (VisualSonics Inc, Toronto, ON, Canada) using a 30 MHz image transducer. Mice were anesthetized with oxygen and isoflurane (1–2%), and systolic function of the left ventricle was obtained from short-axis M-mode scans. Diastolic function was measured using pulsed-wave and tissue Doppler imaging at the level of the mitral valve.

#### 4.2.4. Tail-Cuff Blood Pressure Recordings

Elevated blood pressure is a major risk factor for the development of symptomatic HF [50]. Systolic blood pressure was measured noninvasively in conscious mice using the tail-cuff method (BP-98A). Animals were placed in individual holders on a temperature-controlled platform (37 °C), and recordings were performed under steady-state conditions. Before testing, all mice were trained to become accustomed to short-term restraint. Blood pressure was recorded at least 7 times per mouse, and readings were averaged.

#### 4.2.5. Intraperitoneal Glucose Tolerance Test

An intraperitoneal glucose tolerance test (IPGTT) was performed by injecting glucose (2 g/kg in saline) after 6-hour fasting. Tail blood glucose levels were measured with a glucometer before (0 min) and 15, 30, 45, 60, and 120 min after glucose administration. To determine glucose tolerance, we calculated the area under the curve (AUC).

#### 4.2.6. Exercise Exhaustion Test

Exercise intolerance is common in HF. In the clinic, the 6 min walk test is an alternative way to measure exercise capacity. The distance walked in the 6 min walk test has been associated with prognosis in HF [51,52]. In this study, we used the treadmill-measured exercise capacity of mice in each group. Animals ran on the treadmill starting at a warm-up speed of 5 m/min for 4 min, after which the speed was increased to 14 m/min for 2 min. Every subsequent 2 min, the speed was increased by 2 m/min until the animal was exhausted. Exhaustion was defined as the inability of the animal to return to running within 10 s of direct contact with an electric-stimulus grid. Running time and running distance were recorded when the mice were exhausted.

#### 4.2.7. Histology

Hearts were fixed in 4% paraformaldehyde and embedded in paraffin. Crosswise 5 μm-thick heart sections were cut and stained with hematoxylin and eosin (H&E) (Servicebio, Ghent, Belgium) for routine histological analysis. Masson’s Trichrome (Servicebio) was to evaluate cardiac fibrosis following the manufacturer’s instructions. The slides were then mounted with coverslips using neutral gum (SCRC, 10004160). The images were captured using high-volume, digital whole-slide scanning (Pannoramic MIDI, 3DHISTECH, Budapest, Hungary). Three samples in each experimental group and three sections from each individual sample were analyzed by Image J software.

#### 4.2.8. Statistical Analysis

The experimental data are presented as the mean ± SEM (SEM), and statistical analysis was performed using Graph Pad Prism 5. One-way analysis of variance (ANOVA) was used to evaluate differences between groups, followed by Tukey’s post hoc test. Results with *p* < 0.05 were considered statistically significant.

## Figures and Tables

**Table 1:** The eleven flavonoids obtained from TFDM.

Compound Name	OB (%)	DL	References
Apigenin	23.06	0.21	[17]
Acacetin	34.97	0.24	[12,18]
Luteolin	36.16	0.25	[17]
Salvigenin	49.07	0.33	[17]
Chryseriol	35.85	0.27	[19]
Diosmetin	31.14	0.27	[12]
Isorhamnetin	49.6	0.31	[17]
Kaempferol	41.88	0.24	[19]
Quercetin	46.43	0.28	[17]
Tilianin	19.66	0.79	[14,17]
Astragalin	14.03	0.74	[20]

**Table 2:** The chemical structure of flavonoids in TFDM.

NO	Compound Name	PubChem CID	Structure
1	Apigenin	5280443	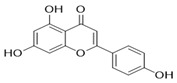
2	Acacetin	5280442	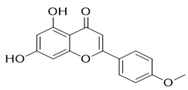
3	Luteolin	5280445	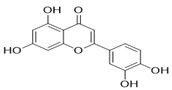
4	Salvigenin	161271	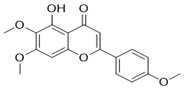
5	Chryseriol	5280666	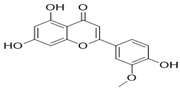
6	Diosmetin	5281612	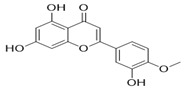
7	Isorhamnetin	5281654	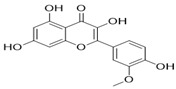
8	Kaempferol	5280863	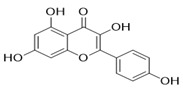
9	Quercetin	5280343	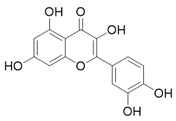
10	Tilianin	5321954	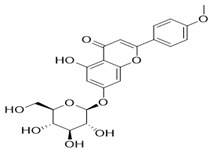
11	Astragalin	5282102	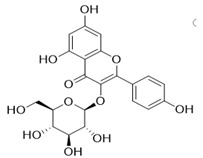

**Table 3:** The top 10 hub genes with higher degree of connectivity.

Gene ID	Gene Name	Gene Symbol	Degree
5743	Prostaglandin-endoperoxide synthase 2	PTGS2	11
4843	Nitric oxide synthase 2	NOS2	10
5742	Prostaglandin-endoperoxide synthase 1	PTGS1	10
367	Androgen receptor	AR	9
5294	Phosphatidylinositol-4,5-bisphosphate 3-kinase Catalytic subunit gamma	PIK3CG	9
7124	Tumor necrosis factor	TNF	8
2099	Estrogen receptor 1	ESR1	7
3569	Interleukin 6	IL6	7
7498	Xanthine dehydrogenase	XDH	7
3553	Interleukin 1 beta	IL-1β	6

**Table 4:** Docking scores (kcal/mol) of compound–target docking.

Compound	TNF	IL-6	IL-1β	NOS2	PTGS2
Apigenin	−8.2	−8.9	−7.3	−10.1	−8.7
Acacetin	−8.2	−8.6	−7.3	−10.2	−8.3
Luteolin	−8.4	−9.2	−7.5	−9.8	−8.6
Salvigenin	−7.9	−8.4	−7.1	−9.1	−8.3
Chryseriol	−8.4	−8.8	−7.3	−9.8	−8.7
Diosmetin	−8.3	−8.7	−7.4	−10.1	−8.4
Isorhamnetin	−8.0	−8.7	−7.2	−9.9	−9.1
Kaempferol	−7.8	−8.8	−7.2	−9.9	−9.0
Quercetin	−8.2	−9.3	−7.4	−9.7	−9.0
Tilianin	−8.0	−9.4	−7.7	−10.9	−9.4
Astragalin	−7.4	−7.6	−7.8	−9.8	−8.7

## Data Availability

All data generated or analyzed during this study are included in this article and are available from the corresponding author on reasonable request.
